# 3D Bi_2_Te_3_ Interconnected Nanowire Networks to Increase Thermoelectric Efficiency

**DOI:** 10.1021/acsaem.1c02129

**Published:** 2021-12-13

**Authors:** Alejandra Ruiz-Clavijo, Olga Caballero-Calero, Cristina V. Manzano, Xavier Maeder, Albert Beardo, Xavier Cartoixà, F. Xavier Álvarez, Marisol Martín-González

**Affiliations:** † Instituto de Micro y Nanotecnología, IMN-CNM, CSIC (CEI UAM+CSIC) Isaac Newton, 8, E-28760 Tres Cantos, Madrid, Spain; ‡ EMPA, Swiss Federal Laboratories for Materials Science and Technology, Laboratory for Mechanics of Materials and Nanostructures, Feuerwerkerstrasse 39, CH-3602 Thun, Switzerland; § Departament de Física, Universitat Autònoma de Barcelona, Campus Bellaterra, 08193 Bellaterra, Barcelona, Spain; ∥ Departament d’Enginyeria Electrònica, Universitat Autònoma de Barcelona, Campus Bellaterra, 08193 Bellaterra, Barcelona, Spain

**Keywords:** thermoelectricity, nanostructure, nanowire, scaffold, bismuth telluride, zT, metamaterial, metastructure

## Abstract

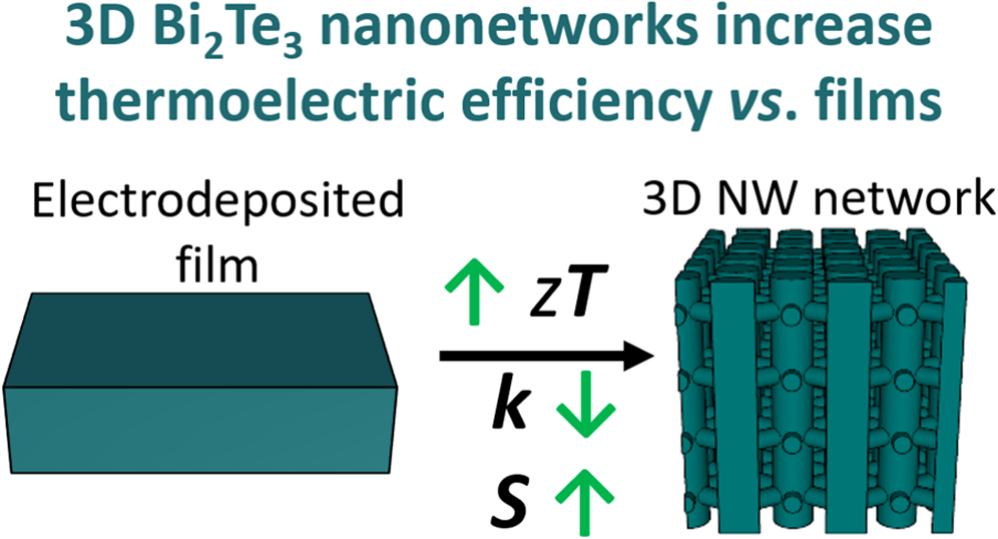

3D interconnected nanowire scaffoldings are shown to increase the thermoelectric efficiency in comparison to similar diameter 1D nanowires and films grown under similar electrodeposition conditions. Bi_2_Te_3_ 3D nanonetworks offer a reduction in thermal conductivity (κ_T_) while preserving the high electrical conductivity of the films. The reduction in κ_T_ is modeled using the hydrodynamic heat transport equation, and it can be understood as a heat viscosity effect due to the 3D nanostructuration. In addition, the Seebeck coefficient is twice that of nanowires and films, and up to 50% higher than in a single crystal. This increase is interpreted as a nonequilibrium effect that the geometry of the structure induces on the distribution function of the phonons, producing an enhanced phonon drag. These thermoelectric metamaterials have higher performance and are fabricated with large areas by a cost-effective method, which makes them suitable for up-scale production.

## 1 Introduction

In recent years, great efforts have been devoted to enhance the thermoelectric efficiency of different materials through nanostructuration. The most popular way was to reduce their lattice thermal conductivity by increasing phonon scattering, either by introducing scattering centers^1^ or by reducing the dimensionality^2^ of the structure. In this way, a significant reduction in the thermal conductivity was obtained when structuring the material in the form of nanowires^3^ compared to bulk or thin-film values. One highly efficient way of obtaining a great number of homogeneous nanowires, as far as composition and diameter are concerned, is via electrochemical deposition inside alumina templates, as it has been shown with bismuth telluride.^4,5^ Nevertheless, such structures present drawbacks such as the difficulties associated with their characterization and implementation in actual devices, given that the alumina template must be dissolved in most cases, which produces the collapse of the nanowire array. In this work, we present a way of overcoming such limitations by nanoengineering the structure in the three dimensions at the nanoscale, thus creating a macro-net of interlinked nanowires.^6^ Such novel structure preserves the advantages of template deposition techniques (reduced cost, easy scalability, dimension reduction, etc.) while exhibiting mechanical stability that enables not only experimental characterization techniques devised for thin films or even bulk samples but also allows easier handling and implementation in devices.^7^ This nanostructure can be understood as a Bi_2_Te_3_-based metamaterial since the structure results in an artificial material with properties that differ from those of bismuth telluride, and which can be tailored as desired by changing the geometry of the alumina template.

As it will be shown in this work, these metamaterials exhibit thermoelectric properties that depend on the geometrical parameters of the nanostructure. In brief, we have obtained an increase in the thermoelectric figure of merit, *zT*, in 3D Bi_2_Te_3_ metamaterials of one order of magnitude compared to Bi_2_Te_3_ nanowires and films prepared under the same conditions. Remarkably, the *zT* value for the 3D metamaterial is higher than in a single crystal measured along the same direction. This increase is due to, both, a reduction in the thermal conductivity even to values below the amorphous limit and a two-fold Seebeck coefficient increase, while preserving a high electrical conductivity similar to the values obtained in the films.

## 2 Materials and Methods

### 2.1 Fabrication Methods

Three-dimensional (3D) Bi_2_Te_3_ nanowire networks or scaffolds were manufactured by electrochemical deposition in 3D anodic aluminum oxide (3D-AAO) templates, as described in ref (8). The fabrication of 3D-AAO templates was developed in 2014, as described in ref (6), and they were fabricated via two-step anodization of 0.5 mm thick aluminum foils (from Advent Research Materials, 99.999%). The first step was performed in a 0.3 M sulfuric acid bath at 0 °C for 24 h and the second, after removing the alumina layer (in a mixture of 7 wt. % phosphoric acid and 1.8 wt.% chromic oxide), combines mild anodization conditions with pulses of hard anodization. After the anodization process, the aluminum substrate and barrier layers were eliminated (using HCl/CuCl_2_ and a 10 wt. % H_3_PO_4_, respectively), followed by etching in 5 wt. % H_3_PO_4_ on the regions where hard anodization was performed, thus opening connections between the longitudinal pores, which were also slightly etched. Hence, structures composed of longitudinal pores interconnected at different heights by transverse nanocanals were obtained. The distance between the centers of adjacent transverse nanocanals is denoted as *P*. In this work, three different structures were fabricated, with *P* = 720, 346, and 220 nm, with an associated error of ±5 nm, corresponding to hard anodization periodic pulses sent each 540, 360, and 180 s, respectively. In all cases, the first region of conventional nanopores of 1 μm long was made before the production of the first transversal nanochannel to increase the stability of the structure.

The 3D-AAO templates were then evaporated with 5 nm of Cr and 150 nm of Au, attaching this surface to a copper holder via silver paste, to form the working electrode of a three-electrode electrochemical cell. Exposed copper was coated with nail polish to electrically isolate the exposed areas, except for the 3D-AAO template. The reference and counter electrodes were saturated Ag/AgCl electrode and platinum mesh, respectively. The electrolyte used consists of 0.9 × 10^–2^ M Bi^3+^ (from Aldrich bismuth pieces, 99.999%), 10^–2^ M HTeO^+^ (from Aldrich tellurium powder, 99.997%), and 1 M HNO_3_ (Panreac 65% nitric acid). Electrochemical growth was performed in pulsed mode, switching from a certain constant deposition potential for 1 s to zero current density for 0.1 s.^9^ The deposition potentials were identified by cyclic voltammetry studies as described in ref (8) to be in the same conditions in all the cases. The depositions and the cyclic voltammetry studies were performed by a bi-potentiostat (Autolab PGSTAT 302) controlled using Nova 1.10 software. For comparison, the same electrodeposition process was performed for more conventional 1D Bi_2_Te_3_ nanowire arrays of the same diameter (55 nm), grown inside a 1D AAO template and Bi_2_Te_3_ films fabricated on a silicon substrate covered with 5 nm chromium and 150 nm gold under electrodeposition conditions optimized to obtain the same orientation and stoichiometries.

Once the 3D-Bi_2_Te_3_ nanowire networks were fabricated inside the 3D-AAO templates, the measurements required the removal of the gold–chromium conductive layer, either by chemical etching (using potassium iodide, KI, and iodine, I_2_, solution in water, in 1:4:4 I_2_/KI/H_2_O ratio for the removal of the Au layer and a Cr etchant consisting of 0.5 M potassium permanganate, KMnO_2_, and 0.5 M sodium hydroxide, NaOH, compatible with Bi_2_Te_3_), either by polishing the surface with a suspension of alumina powders of different sizes (5, 1, 0.3, and 0.05 μm, in this order, collected from Buehler). When needed, the 3D-AAO template was removed by immersing the samples in a solution of 7 wt. % H_3_PO_4_ and 1.8 wt. % CrO_3_ for 24 h to leave the 3D, free-standing, Bi_2_Te_3_ nanowire networks.

### 2.2 Measurement Techniques

**Figure 1:**
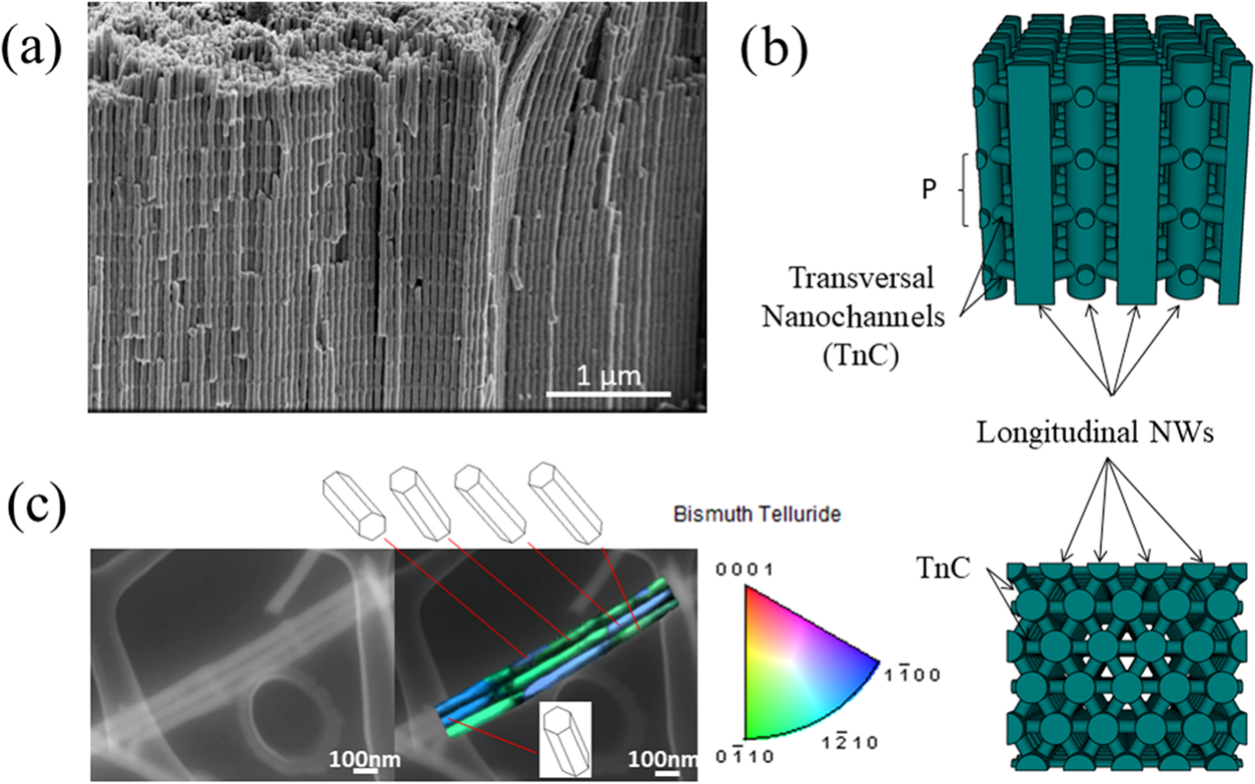
(a) Scanning electron microscopy (SEM) image of a free-standing bismuth telluride scaffold metamaterial obtained after the dissolution of the AAO template. In this image, the transverse interconnections are 220 nm apart (this distance is named as *P* in (b)). (b) Schematic 3D representation of the scaffold composed of perpendicular interconnected nanowires in lateral view (top), like what can be observed by SEM, and top view (down), where the hexagonal distribution of the longitudinal nanowires and the interconnections between neighbors can be seen. (c) SEM image and the transmission electron backscatter diffraction (t-EBSD) of a set of three connected nanowires, showing a clear orientation of the *c*-axis perpendicular to the growth direction in all of the structures.

The samples were prepared according to the measurement method, as shown in Figure S1. Morphological investigations were carried out using a high-resolution scanning electron microscope (HRSEM, FEI Verios 460). The quantitative compositions of the samples were studied using energy dispersive X-ray spectrometry (EDX Hitachi S-3000 from SIdI-UAM). The preferential crystalline orientation and the material phase were measured by X-ray diffraction using a Philips X’Pert PANalytical four circles diffractometer with Cu K_α_ wavelength of 0.15418 nm. All of these characterizations were made on pristine samples without additional preparation (Figure S1a). Then, the 3D-AAO template was dissolved for certain samples resulting in self-sustained structures, such as that shown in Figure 1a. They were then sonicated to obtain arrays of tens of nanowires connected with the transversal nanocanals. The resulting 3D Bi_2_Te_3_ nanowire networks were carefully dispersed on carbon grids avoiding structural bending or twisting (see Figure 1c), for electron tomography measurements and transmission electron backscatter diffraction (t-EBSD).^10^ The transmission Kikuchi diffraction (TKD) analysis along the 3D nanostructure was performed using a Tescan MIRA SEM instrument (TESCAN, Czech Republic), with beam conditions of 30 kV and 5 nA, using a tilt of 20° and 3 mm of working distance. TKD crystal orientation maps were made using step sizes from 5 to 15 nm.

For the characterization of the transport properties (Seebeck coefficient and electrical conductivity) along the in-plane direction, that is, parallel to the surface of the 3D-Bi_2_Te_3_ nanowire network (and along the direction of the transversal canals), the gold–chromium layer had to be removed before the measurements, and in most cases, silver paint contacts were made in the polished surface (see Figure S1c). The in-plane Seebeck coefficient was obtained at room temperature in a lab-made measurement system where an in-plane thermal gradient can be established. Then, both the actual temperature difference Δ*T* and voltage difference Δ*V* set at two different points of the samples were measured, and the Seebeck coefficient *S* = Δ*V*/Δ*T* was obtained. The in-plane electrical conductivity measurements were performed in a four-probe system based on a Van der Pauw configuration (a commercial HMS-5500 Hall Effect Measurement system from Ecopia). Moreover, the Seebeck coefficient measurements of the out-of-plane configuration were also performed in a lab-made system consisting of two blocks of copper between which the sample was sandwiched, similar to those reported in ref (11). Then, the temperature of one of the copper blocks could vary around room temperature and the voltage on both sides of the sample was measured. For these measurements, the 3D-Bi_2_Te_3_ nanowire networks had to be filled, given that electrical contact between both sides of the sample was necessary. In this case, the sample’s surface was polished until the tip of the 3D nanonetwork was reached (see Figure S1b in the Supporting Information).

The out-of-plane thermal conductivity measurements were performed using a lab-made system based on the Photoacoustic effect, as described in refs (12, 13), which is systematically tested with different calibration samples to guarantee the accuracy of the measurements. To perform the measurement, after evaporating 80 nm of titanium in the surface where the gold and chromium layer are (see Figure S1.d in the Supporting Information), the sample is introduced in a custom-designed Photoacoustic cell. It is then heated with a pulsed laser (980 nm wavelength Alphalas fiber LDF-10 laser) with the titanium layer acting as a transducer to maximize laser absorption. The heating of the sample causes an expansion in the air in contact with the surface of the sample, which is transferred as acoustic waves in the cell, and recorded with the aid of a microphone (G.R.A.S. 46 BL 1/4″CCP pressure microphone). Then, from the phase difference between the incident laser and the acoustic wave, which are recorded thanks to a Lock-in Amplifier (Signal Recovery Model 7270 DSP), the thermal diffusivity can be obtained using a multilayer model developed by Hu et al.^14^ Finally, by comparing the signal of the problem with a reference (quartz with an 80 nm Ti layer) and knowing the density and the specific heat, thermal conductivity can be obtained.

## 3 Theory and Calculation

### 3.1 Electrical Conductivity of the 3D Structure

To model the effective electrical resistivity of the 3D-Bi_2_Te_3_ nanowire networks, we used a COMSOL model of the system (shown in Figure S2a). The intrinsically anisotropic electrical conductivity was taken from the measurements in electrodeposited films^15^ and introduced in the electrical conductivity tensor of the material. Then, the steady-state electric potential for a system of *N* nanowires was simulated, imposing a potential difference in two different nanowire terminals, in analogy with experiments (see Figure S2b,c for the results obtained in two different structures when modifying the number of nanowires and thus increasing the distance between the terminals).

According to the model, the main contribution to the total in-plane resistance of the simulated structure is located on the transversal canals, and the contribution obtained in the nanowires can be neglected. Specifically, the results show that the total in-plane resistance of the structure is approximated by *R* ≃ ρ_TnC_·*d*/(*A*_TnC_*·n*), where ρ_TnC_ is the intrinsic resistivity in the direction of the transversal canals, *d* is the distance between the terminals, *n* is the number of canals (which can be determined experimentally for each sample from SEM images of the lateral view of the structures), and *A*_TnC_ is the transversal area of one canal. This is due to the smaller intrinsic resistivity in the nanowire direction (out-of-plane) along with *d* being much larger than the thickness of the considered structures. Consequently, note that the total resistance does not explicitly depend on the distance between canals, *P*.

### 3.2 Hydrodynamic Heat Transport Equations and Boundary Conditions

In this section, we show the complete system of partial differential equations, boundary conditions, and intrinsic material properties required for predicting the heat flux *q* and the temperature *T* both in the semiconductor and the oxide domains in steady-state conditions using COMSOL Multiphysics. Only a single periodically repeated geometry cell is simulated. We also show here the inclusion of the electron contribution to the thermal conductivity.

#### 3.2.1 3D Bi_2_Te_3_ Interconnected Nanowire Network

Heat conduction in the semiconductor domains is described using the Kinetic Collective Model (KCM), consisting of the energy conservation and the hydrodynamic heat transport equations in steady state^16−18^
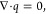
1
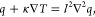
2where κ = 2.3 W·m^–1^·K^–1^ is the Bi_2_Te_3_ bulk lattice thermal conductivity and  is a weighted phonon mean free path average, known as nonlocal length.^19^

For illustration, we focus first on the non-Fourier behavior obtained in the semiconductor and we do not include in the simulations the oxide matrix. Therefore, in all the boundaries except in the nanowires (NWs) and the transversal nanocanals (TnCs) terminals, we impose thermal insulation
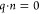
3and the slip boundary condition^17^
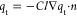
4where *n* points away from the material and subindex t denotes the tangent-to-the-surface heat flux component. The slip coefficient *C* depends on the fraction of specular phonon reflections on the boundary, as discussed in ref (17). Here we assume diffusive boundary reflections so that *C* = 1. To calculate the effective thermal conductivity along the NW direction (out-of-plane), we fix heat flux periodic boundary conditions with imposing a temperature difference Δ*T* = 1 K in the NW terminals, and we impose periodic boundary conditions for the heat flux and the temperature in each pair of opposing TnC terminals.

From the resulting stationary solutions, we calculate the effective lattice thermal conductivity of isolated Bi_2_Te_3_ structures as
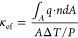
5where Δ*T*/*P* is the temperature gradient imposed along the NW direction (out-of-plane) and *A* is the cross-sectional area transversal to the temperature gradient.

κκ and *l* from eq 2 are intrinsic parameters that do not depend on the geometry. Therefore, the same parameter values can be used to predict the lattice thermal conductivity of isolated Bi_2_Te_3_ (without the TnCs) reported in ref (4) (see Supporting Information Figure S5).

Note that here we assumed an isotropic thermal conductivity κ in eq 2, corresponding to the largest component of the bulk anisotropic lattice thermal conductivity tensor. This component also corresponds to the direction of the temperature gradient imposed in experiments, which is the longitudinal nanowire direction (out-of-plane). To improve the accuracy of the predictions, an anisotropic version of the hydrodynamic heat equation is required. However, we expect the resulting correction to be small because the heat flux mainly flows in the nanowires’ longitudinal direction (out-of-plane). Moreover, note that predicting the effective thermal conductivity in the in-plane direction, that is, along the TnCs direction, using the presented isotropic model, requires the use of the adequate lattice thermal conductivity tensor component (κ = 0.9 W·m^–1^·K^–1^). In this case, the temperature difference is imposed in two opposed TnC terminals, which also implies modifying the cross section for integration in eq 5. The steady-state heat flux profile according to the hydrodynamic model for a free-standing network when the temperature gradient is imposed in the in-plane direction can be seen in Figure S3 in the Supporting Information.

#### 3.2.2 Inclusion of the Oxide Matrix

To compare theory and experiments, the semiconductor structure is embedded in an oxide matrix. The oxide domains fill all the space that is not occupied by the Bi_2_Te_3_ network in each periodically repeated cell. Since the phonon mean free paths in the oxide are much smaller than the geometry characteristic sizes, nonlocal effects are not expected, and Fourier’s law can be used to describe these domains
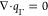
6
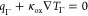
7where κ_ox_ = 1.25 W·m^–1^·K^–1^ is the oxide bulk thermal conductivity. Subindex Γ denotes the temperature and the heat flux in the oxide domains.

In the oxide terminals contiguous to the NW terminals, we impose periodic heat flux boundary conditions along with the corresponding temperature difference Δ*T* = 1 K. Similarly, in the oxide faces contiguous to opposed TnC terminals we impose periodic boundary conditions for the heat flux and the temperature. Finally, instead of the insulation boundary condition (eq 3), we impose continuity of the heat flux normal component in the oxide-semiconductor interfaces
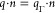
8It is worth noting that the slip boundary condition (eq 4) is still required to model the heat flux tangential component in the semiconductor domain. Conversely, this boundary condition is not required on the oxide side.

From the resulting stationary solutions, we calculate the effective lattice thermal conductivity of the Bi_2_Te_3_ structures embedded in the oxide matrix using expression (eq 5), considering both the area covered by the NW terminal and by the oxide terminal.

#### 3.2.3 Electronic Contribution to the Total Thermal Conductivity

**Figure 2:**
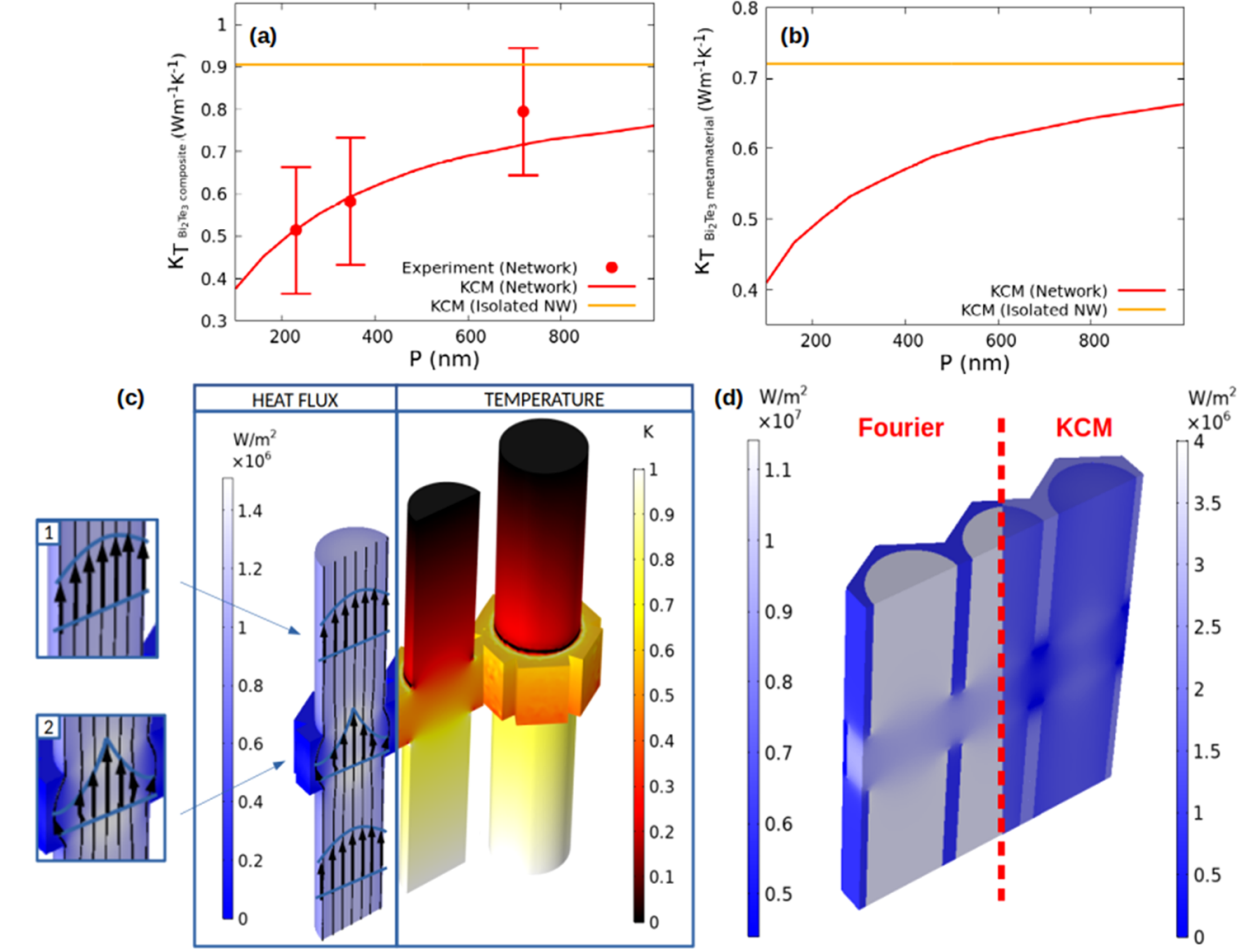
(a) Thermal conductivity in the out-of-plane direction predicted by the hydrodynamic heat transport model (KCM), considering the lattice and the electronic contributions, as well as the 3D-AAO matrix, along with the experimentally obtained values. The red line represents the thermal conductivity of the composite obtained by the model, and the red points correspond to the measured thermal conductivities obtained for different values of *P*. The orange line represents the predicted value for a 55 nm diameter Bi_2_Te_3_ nanowire array embedded in the alumina matrix. (b) Thermal conductivity in the out-of-plane direction (κ_T_) predicted by the hydrodynamic heat transport model, considering the lattice and the electronic contributions, for the self-supported bismuth telluride metamaterial scaffold, excluding the matrix (red line). The orange line represents the predicted value for a 55 nm diameter nanowire without the alumina template. (c) Heat flux and temperature steady-state profiles according to the hydrodynamic heat transport model for a free-standing 3D network. Two different nonlocal effects reducing the effective thermal conductivity of the structure can be observed: the inhomogeneous heat flux profile in the nanowire (inset 1) and the curvature of the heat flux streamlines in the regions connected by the TnCs (inset 2) when the heat is along the nanowire direction. (d) Heat flow in the 3D Bi_2_Te_3_/alumina composite according to the hydrodynamic heat transport model (KCM) and the (bulk) Fourier approach. Additional information of the thermal conductivity in the in-plane direction predicted by the KCM can be found in the Supporting Information.

In the previous subsections, we presented the model required to predict the lattice thermal conductivity of the structures. To compare with the experimentally measured thermal conductivity in Figure 2, the contribution of electrons must be added to the Bi_2_Te_3_ lattice conductivity. Using the measured electrical conductivity of the Bi_2_Te_3_ NWs σ from ref (20) and the Wiedemann–Franz law
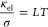
9where *L* = 2.44 × 10^–8^ W·Ω·K^–2^, one can calculate the electronic thermal conductivity, κ_el_. Consider the present case of an NW diameter *D* = 55 nm. The corresponding electrical conductivity in the out-of-plane direction (perpendicular to the c-axis) is σ = 3 × 10^4^ S/m^2^.^20^ The out-of-plane electrical conductivity of the network in the direction of the NW is the same as in the isolated NW (the TnCs are not expected to influence electronic transport). Therefore, the electronic thermal conductivity in all the cases under study is κ_el_ = 0.22 W·m^–1^·K^–1^. Finally, the electronic thermal conductivity is weighted by the areal density *x* covered by the NWs for the full structure including the oxide so that the contribution of the electronic thermal conductivity is *x*·κ_el_ = 0.14 W·m^–1^·K^–1^. The sum of the lattice and the electronic contribution to the total thermal conductivity is presented in Figure 2a (including the oxide matrix) and Figure 2b (without the oxide). Analogous calculations using the experimentally measured electrical conductivity in the in-plane direction are done to calculate the electronic contribution to the thermal conductivity in the direction of the TnCs (i.e., in-plane).

## 4 Results and Discussion

The bismuth telluride metamaterials prepared for this study (shown in Figure 1) comprise a three-dimensional arrangement of longitudinal nanowires of 55 nm in diameter connected by transversal nanowires, also referred here as interconnections, or nanocanals (TnC), with approximate dimensions of 40 nm in height and 30 nm in width. The previously tailored 3D porous alumina (3D-AAO) templates or matrices, in which the nanowires are deposited precisely controlled the distance at which the connections are formed into.^6^ We determined the best conditions to obtain nanowires highly oriented along [110] (see Figure S4 in the Supporting Information), with the exact Bi_2_Te_3_ stoichiometry and high crystallinity, by electrodeposition into the 3D porous alumina templates. The metamaterial obtained can be made free-standing without destroying its structure (see Figure 1a) by selectively dissolving the alumina template, because the interconnections hold the 3D scaffold together. Since the electrodeposition allows the fabrication over large areas, and the fabricated templates at the lab scale are several cm^2^, the resulting metamaterial can be measured using conventional thin-film techniques.

We measure one order of magnitude increase in *zT* of the bismuth telluride metamaterial versus films and 1D nanowires (grown by the same technique and of similar composition, and orientation), due to both, a reduction in the thermal conductivity and an increase in the Seebeck coefficient of the nanowire network. The electrical conductivity (σ) remains similar in 3D Bi_2_Te_3_ than that of nanowires and films (3.0 × 10^4^ and 6.7 × 10^4^ S·m^–1^, respectively; see Table 1). The thermal conductivity measured by the Photoacoustic technique^13^ along the length of the nanowires (out-of-plane direction) is reduced to values as low as κ_T_ = 0.5 W·m^–1^·K^–1^. This is lower than in highly oriented electrodeposited films, which showed a value of κ_T_ = 2.2 W·m^–1^·K^–1^ at room temperature when measured perpendicular to the c-axis (out-of-plane direction in [110] oriented films). It is also lower than that of nanowires of 52 ± 5 nm in diameter, where a thermal conductivity of κ_T_ = 0.72 ± 0.37 W·m^–1^·K^–1^ has been reported when measured along the nanowire length (out-of-plane).^4^ It is worth noting that all these measurements were carried out at room temperature using the same technique. Similarly, the value of the Seebeck coefficient presents a two-fold increase compared to values reported in the literature for stoichiometric and oriented nanowires fabricated by electrodeposition, that range between −30 and −57 μV·K^–1^.^21−23^ In contrast, for the bismuth telluride metamaterials reported here, the Seebeck coefficient is as high as −127 ± 6 μV·K^–1^ when measured in-plane.

**Table 1:** Comparison of the Reported Values of Bulk,^31^ Electrodeposited Films, and Nanowires with the 3D Bi_2_Te_3_Metamaterial Presented in This Work^a^

	Structural information: period (*P*) and diameter (*D*) in nm	Seebeck (μV·K^–1^) ∥*c*-axis except indicated	σ (S·m^–1^) ∥ *c*-axis except indicated	κ_T_ (W·m^–1^·K^–1^) (calculated from measurements) ⊥ *c*-axis	κ_T_ (W·m^–1^·K^–1^) (calculated from the model) ∥ *c*-axis	calculated zT* and direction
Bi_2_Te_3_ 3D nanonetwork metamaterial	*P* = 220 & *D* = 55	–127 ± 6	(7.1±0.6) × 10^4^	0.501	0.62	0.55 ∥*c*-axis
						
	*P =* 346& *D* = 55	–103 ± 5	(6.6±0.6) × 10^4^	0.554	0.58	0.36 ∥*c*-axis
						
	*P =* 720& *D* = 55	–121 ± 6	(9.8±0.9) × 10^4^	0.632	0.81	0.53 ∥*c*-axis
						
Bi_2_Te_3_ nanowires^4,20^	*D* = 55	–55 ⊥ *c*-axis measured in the out-of-plane direction	3.0 × 10^4^ ⊥ *c*-axis measured in the out-of-plane direction	0.72		0.038 ⊥*c*-axis
						
Bi_2_Te_3_ thin film^15^	∥*c*-axis	–58	6.7 × 10^4^		1.7	0.04 ∥*c*-axis
						
	⊥*c*-axis	–48	1.4 × 10^4^	2.2		0.004 ⊥*c*-axis
						
Bi_2_Te_3_ single crystal^31^	∥*c*-axis	–240	1.8 × 10^4^		1	0.32 ∥*c*-axis
						

a zT^*^ is reported for room temperature. Seebeck and electrical conductivities have been measured in the in-plane configuration (and thus, ∥c-axis) unless otherwise mentioned in the table. The thermal conductivity values are the sum of the electronic and phononic contributions as calculated from the hydrodynamic model without considering the oxide matrix (see the Section 2).

To understand the reduction in thermal conductivity, metamaterials with three different distances between transversal interconnections (namely, *P* = 720, 346, and 220 nm) were studied to see if the reduction was related to this geometrical parameter. The thermal conductivity values for the different periods are shown in Figure 2 (and also in Table S1 in the Supporting Information), along with those obtained for 1D Bi_2_Te_3_ nanowires. Specifically, Figure 2a shows the thermal conductivity for the different *P* of the composite Bi_2_Te_3_/alumina and Figure 2b shows the conductivity of the free-standing 3D nanonetworks (see the Section 2). Both the lattice and the electron contribution to the total thermal conductivity were considered in the simulations. As it can be seen, by comparing the results of 1D nanowire arrays of similar diameter (orange line in Figure 2a,b) to that of the 3D Bi_2_Te_3_ scaffolds inside the 3D porous alumina, the measured thermal conductivity is lower in the metamaterial (red curve). Furthermore, this reduction is greater when the intercanal distance (*P*) is reduced, indicating that the reduction is associated with the transversal interconnections. The physical reasons for this reduction in thermal conductivity can be explained by the heat transport eq 2, known as the Guyer–Krumhansl equation (GK), which generalizes Fourier’s law by including nonlocal effects.^16−18^ This equation predicts a heat flux reduction in a region of size *l* near the Bi_2_Te_3_ nanowire surface due to phonon-boundary scattering^17^ that has its origin in the Laplacian term of the GK equation. By analogy to classical fluid mechanics, this term can be interpreted as a heat viscosity effect. Accordingly, the effective thermal conductivity of electrodeposited 1D nanowires is reduced in comparison to electrodeposited thin films^10^ because collisions reduce the heat flux in a region near the wire boundary (see Figure 2c, inset 1, and Supporting Information Figure S5, where the predicted and measured lattice conductivities are displayed for different 1D nanowire diameters). Alternatively, according to a kinetic interpretation, the reduction of the effective thermal conductivity in the wire can be attributed to a suppression of phonons with a mean free path larger than the diameter of the wire. Considering that *l* is an integral expression over the phonon mean free paths,^19^ both approaches can provide similar predictions for the influence of the boundaries in this case.

Furthermore, according to eq 2, there is an additional and enhanced viscous effect on the 3D networks due to the inhomogeneities in the heat flux profile (i.e., an enhanced heat flux Laplacian) originated in the regions where transversal and longitudinal nanowires are connected (see Figure 2c, inset 2). This extra source of heat viscosity, compared to the conventional 1D nanowire geometry, is responsible for the further reduction of the thermal conductivity in the networks. This explains why by increasing the number of interconnections (that is, by reducing *P*), the reduction in thermal conductivity is enhanced, as shown in Figure 2a,b. This effect of the network nanostructuration is qualitatively consistent with non-equilibrium molecular dynamics simulations in single-crystal 3D silicon networks.^100^ Notice that this effect cannot be predicted using the kinetic interpretation in terms of phonon suppression. In the regions where the wires are interconnected, the Bi_2_Te_3_ domain size increases, and thus, the effective thermal conductivity should be increased due to a reduced number of suppressed phonons in those regions. However, the experimental thermal conductivity of the system is reduced in the network as predicted by the hydrodynamic model.

It is interesting to note that, if the alumina is not dissolved from the structure, the viscous effects in the 3D nanonetwork structure dramatically affect the Bi_2_Te_3_/alumina relative contribution to the effective lattice thermal conductivity of the composite (see Figure 2d). The COMSOL solutions show that, according to the bulk Fourier description (i.e., assuming  = 0), heat flows preferentially through the nanowires. However, when including the hydrodynamic effects that block heat flow inside the 3D nanonetworks (i.e.,  = 55 nm), the heat flows preferentially through the alumina, since the effective lattice thermal conductivity of the 3D network becomes smaller than that of the amorphous alumina. Remarkably, the resulting total effective thermal conductivity of the composite formed by the alumina and the Bi_2_Te_3_ interconnected nanostructure as a function of the period of the nanostructure, *P*, is in good agreement with the experimentally obtained data as shown in Figure 2a.

In the case of the total thermal conductivity (κ_T_) in the perpendicular direction, so parallel to the c-axis and the transversal nanocanals (in-plane direction), a value between 0.6 and 0.8 W·m^–1^·K^–1^ can be calculated for the three periods studied in this work. This value is ∼2.5 times lower than the value measured in electrodeposited Bi_2_Te_3_ films along the same direction (see Table 1). In this direction, the viscous effects at the interconnections are significantly larger than along the longitudinal wire-axis direction due to the smaller size and interdistance of the TnCs. This reduces the lattice contribution to κ_ph_ ∼0.1 W·m^–1^·K^–1^ (see the Section 2), a value that is significantly smaller than the electron contribution κ_el_ ∼ 0.6 W·m^–1^·K^–1^. At this point, κ_el_ + κ_ph_ ≃ κ_el_, and the Wiedemann–Franz law allows the simplification of
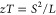
10where *L* is the Lorentz number. This opens a new research venue since *zT* only depends on the Seebeck coefficient and the Lorentz number.

Interestingly, the reduction in thermal conductivity reported for 3D nanowire networks compared with the electrodeposited films is not observed in the electrical conductivity. The values obtained for the electrical conductivity are similar when measured along the same direction (6.7 × 10^4^ S·m^–1^). For the electrical conductivity, a Van der Pauw method was used, taking advantage of the morphology of the metamaterial, which enables the measurement with thin-film techniques. The metamaterial may be considered in the in-plane direction as thin layers with triangular nanoholes that are interconnected through the longitudinal nanowires. The value measured experimentally is the resistivity of the sheet, shown in Table SI in the Supporting Information along with the total thickness of the metamaterial samples. We solved the Laplace equation for the electrical potential considering the actual geometry of the metamaterial and the anisotropy in the intrinsic electrical resistivity of electrodeposited bismuth telluride using COMSOL Multiphysics (see the Section 2 and Supporting Information Figure S2). The solutions show that the potential drop in our structures is localized in the interconnecting channels. Therefore, as the COMSOL model shows, the electrical resistivity is obtained by multiplying the measured sheet resistivity by the number of canals interconnecting two nanowires times the thickness of one canal. The resulting electrical conductivity in the Bi_2_Te_3_ nanoscaffolds (see Table 1) is of the same order of magnitude as the electrodeposited thin film oriented in the same direction, that is, 6.7 × 10^4^ S·m^–1^, as reported in ref (15). Thus, the electrical conductivity appears to be unaffected by the nanostructuration along the *c*-axis. Nonetheless, it should be noted that for the same total thickness of a 3D bismuth telluride-based metamaterial, the lower the *P*, the lower the total electrical resistance.

The other main difference of the Bi_2_Te_3_ 3D nanowire networks versus electrodeposited thin films or nanowires is the increase in the measured Seebeck coefficient. In nanocrystalline, stoichiometric, and strongly oriented films grown by the same pulsed deposition method to be used for comparison in this work, a Seebeck coefficient of −58 μV·K^–1^ has been measured. For stoichiometric, one-dimensional, 55 nm diameter Bi_2_Te_3_ nanowires also fabricated in this work, the Seebeck values measured in the nanowire direction (out-of-plane) are of the same order of magnitude, around −55 ± 3 μV·K^–1^. These are among the best values for this nanowire diameter reported in the literature (−30 μV·K^–1^ to −57 μV·K^–1^). However, in 3D Bi_2_Te_3_ metamaterial produced similarly as films and 1D nanowires, we have measured Seebeck values in the in-plane direction which range from −103 ± 5 to −127 ± 6 μV·K^–1^ (see Table 1). These are more than twice the value found in 1D nanowires or electrodeposited films prepared under similar conditions. It must be stated that no further thermal treatment has been performed on the 3D scaffoldings to be compared to the 1D 55 nm nanowire template and films. Moreover, note that the carrier concentration is similar in films, nanowires, and the 3D nanostructured metamaterial. Hence, the influence of the specific network geometry must be accounted for to interpret the measurements.

This startling increase in the Seebeck should be attributed again to the interconnecting nanocanals. An educated explanation of the increase in the Seebeck coefficient in the 3D nanonetwork versus nanowires and films can be obtained by considering that the Seebeck coefficient has two contributions: One related to the form of the energy band and a second one because of the transfer of momentum between phonons and electrons (phonon drag contribution). The latter is significant when the system is far from equilibrium. Traditionally, this contribution has been observed at low temperatures, where two combined effects take place. On the one hand, the average mean free path of the phonons increases, and normal (momentum-conserving) collisions are more abundant. These two combined effects cause the phonon distribution function to be displaced from the local equilibrium because of the existence of a conserved momentum that cannot be relaxed. In other words, the phonon distribution should populate the different modes with the constraint of preserving the momentum. The consequence of this situation on the thermoelectric effect is that, when the electron cloud interacts with the phonons, it absorbs part of this momentum through electron-phonon collisions. Therefore, the electron distribution is also displaced from local equilibrium and the electrons get momentum in the phonon’s flux direction, thus causing the increment in the Seebeck coefficient.

Even though the usual point of view is that low temperatures are needed to observe the phonon drag contribution, one must consider the more general condition to unlock this effect: The phonon system should be far from equilibrium due to the conservation of momentum in a certain region with an established temperature gradient. The hydrodynamic model used to describe the thermal behavior of our samples shows that this far-from-equilibrium situation can also be observed at room temperature. The key point is the complex nanostructuration of the sample.

From a microscopic point of view, the phonon drag appears because the distribution function of the phonons is not the equilibrium one. On the one hand, at low temperatures and under a homogeneous temperature gradient ∇*T*, the effect of the normal collisions cause the distribution to adopt the form , where  is the phonon wavevector and  is a parameter depending on ∇*T*. On the other hand, in the presence of inhomogeneities and regardless of the abundance of normal collisions, it has been shown that a more general nonequilibrium distribution can be proposed
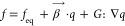
11where  and *G* depend on the phonon velocity and mean free path.^19^ Accordingly, the appearance of a spatially varying heat flux drives the system out of equilibrium. Therefore, in the regions where the heat flux has a larger spatial variation, the phonon system will be far from equilibrium, and phonon drag can emerge. Notice that, contrary to the normal-collision-related phonon drag, which can only be enhanced by reducing the temperature, this new effect can be tailored by including obstacles to the heat flux to make it follow a trajectory with continuous contractions and expansions as in the 3D network.

**Figure 3:**
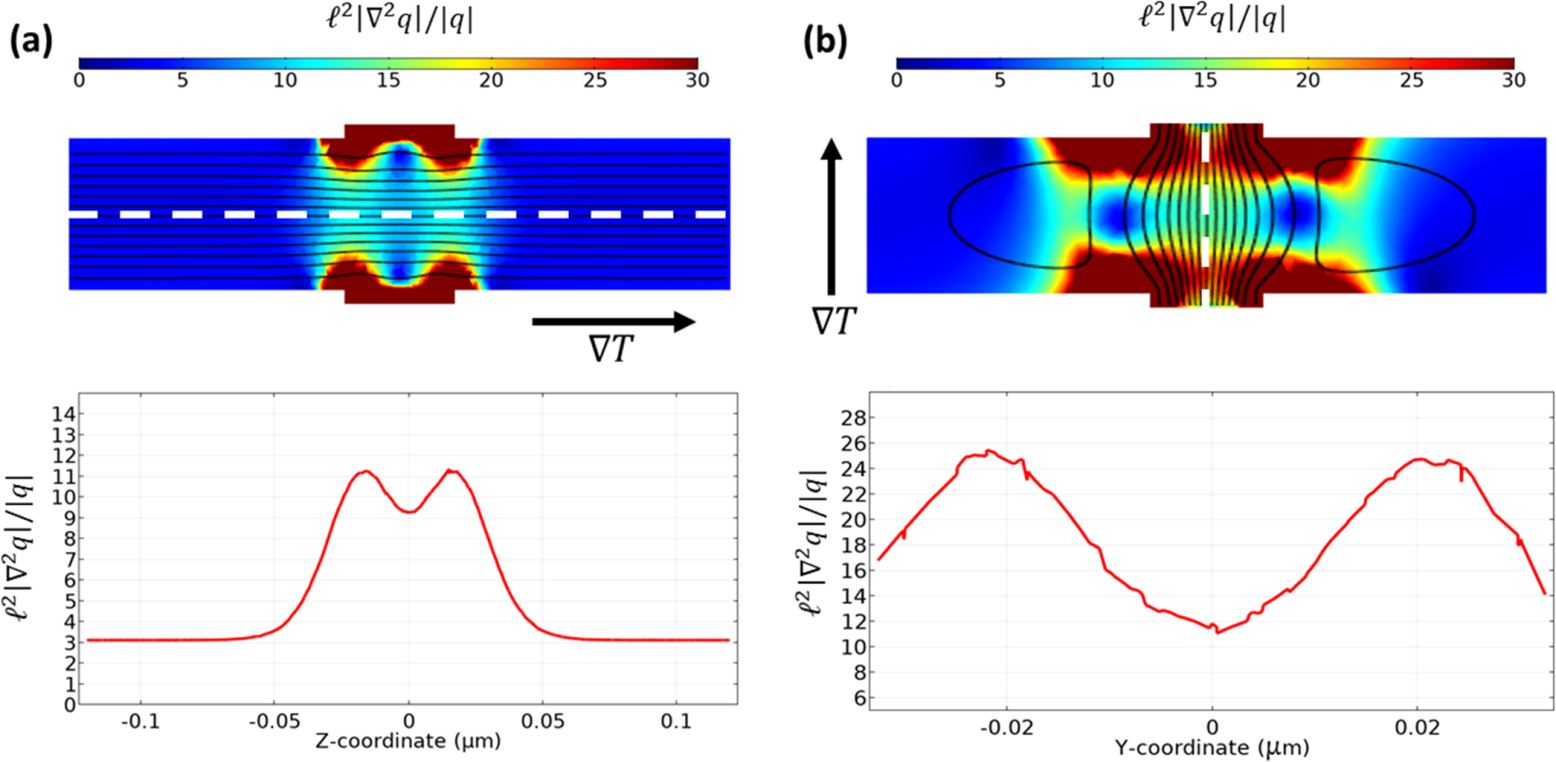
Top (color): Ratio between the Laplacian term and the heat flux term inside the structure in the situation where the temperature gradient is applied along the out-of-plane direction (a) and the in-plane direction (b) (the temperature gradient direction is indicated by a black arrow). The phonon flux streamlines are indicated in black in both cases. Bottom: Value of the ratio between the Laplacian and the heat flux term across the white dash line for both cases.

Using the Boltzmann transport equation and eq 11, it can be shown that the appearance of these nonequilibrium effects is associated with the Laplacian term in the transport eq 2.(19) Therefore, a local measure of the deviation from equilibrium is the quotient between the Laplacian term, *l*^2^∇^2^*q*, with respect to the flux term *q*. In our samples, the increase of the Laplacian term is not due to an increase of the normal collisions, but it is a consequence of the sample characteristic sizes, which are comparable to the mean free path of the resistive collisions. Under such conditions, the momentum is only efficiently destroyed in the boundaries, and the phonon momentum cannot be relaxed inside our samples, which might increase the phonon drag contribution. In Figure 3, we have represented the quotient between the Laplacian and the flux terms in the situations where the heat is flowing in the longitudinal wire direction (out-of-plane), and where the heat flows in the TnCs direction (in-plane). The direction of the flow is indicated by the dashed white line, and the corresponding simulated phonon flux is shown in black lines. Notice that, while in the out-of-plane direction the value of the quotient is ∼3 (except close to the connection with the TnCs where it grows), in the in-plane direction this quotient is an order of magnitude larger (∼20 on average). Therefore, we hypothesize that the observed increase in the Seebeck coefficient in the 3D network (measured in-plane) with respect to the 1D nanowires (measured out-of-plane) may be due to the emergence of a significant phonon drag contribution due to an enhanced deviation from equilibrium in the network. Further work should be done to connect this phenomenological explanation with more fundamental microscopic models providing numerical expressions for the Seebeck coefficient.

Additionally to the previous explanation, it should not be ruled out that there are other possible sources of the increase in the measured Seebeck coefficient, which might come from the increase in crystallinity of the 3D nanostructures and nanowires compared with electrodeposited films oriented in the same direction. Or, even the reduction or disappearance of the effect of topological surface states due to the smaller diameter of the nanocannals than the diameter of the nanowires in our 3D interconnected networks.

In the first case, it has been observed that the crystallinity in the 3D nanostructured metamaterial and the nanowires are higher than in Bi_2_Te_3_ films grown by electrodeposition. This can be seen from the comparison of the crystallite size of electrodeposited thin films, where from the X-ray diffraction data (shown in Supporting Information Figure S4.d) a crystallite size of 37.6 nm is obtained using the Scherrer equation. In the case of 3D Bi_2_Te_3_ nanowire networks, the crystallite size obtained from the X-ray diffraction data ranges between 35 and 40 nm, which is in the order of the size of the TnCs. Nevertheless, as it can be seen in Figure 1c, t-EBSD measurements show that the 3D nanowire networks maintain the same orientation through hundreds of nanometers along the length of the longitudinal nanowires, showing a much higher crystallinity than the films. Taking into account that it has been reported that the increase in crystallinity increases the Seebeck coefficient, this crystallinity enhancement might also contribute to the Seebeck enhancement found in these samples. For instance, in Bi_2_Te_3_ when the samples are annealed^24^ or have a larger grain size,^25^ the Seebeck coefficient can increase up to ten-fold.^24^ In the second case, it cannot be ruled out the influence of the topological surface states, which are known to be detrimental to the Seebeck coefficient. These surface states can be affected by the reduction in diameter of the transverse nanocanals (around 35 nm) compared to the diameter of the nanowires (55 nm). Indeed, this has been experimentally observed in closely related systems, such as Sb_2_Te_3_ thin films^26^ or Bi_1–*x*_Sb*_x_* nanowire arrays.^27^ In particular, it is shown in ref (27) that the magnitude of the Seebeck for Bi_0.85_Sb_0.15_ NWs increased a factor of ∼2 when their diameter was reduced to 41 nm with respect to the minimum Seebeck for a diameter of 58 nm. This reduction in the detrimental character of the surface states was attributed to their hybridization, where the bulk-extending tails of surface states from opposing boundaries overlap. This opens a gap in the surface states, reducing the carrier concentration in the surface states (SS) and increasing the Seebeck coefficient. Another possible source of gap opening is that, in an NW configuration, surface states must be antiperiodic when circling the NW perimeter, thus leading to gap formation.^28−30^

Overall, the performance of the bismuth telluride metamaterial exceeds that of electrodeposited thin films and nanowires of similar diameter by more than an order of magnitude (see Table 1). Moreover, it should be noted too that the calculated *zT* values of the 3D Bi_2_Te_3_ metamaterial can be 50% higher than bulk single-crystalline Bi_2_Te_3_ measured along the same direction (parallel to the c-axis).

## 5 Conclusions

In conclusion, we presented a novel metamaterial based on bismuth telluride, which is a nanostructured material whose properties depend on the geometrical parameters of the 3D structure. The fabrication technique, based on anodization and electrochemical deposition, is economical and easily scalable. Thanks to the nanostructuration, this thermoelectric metamaterial has a reduced thermal conductivity and an improved Seebeck coefficient compared to thin films and nanowires produced and measured under similar conditions. Both parameters enable an enhancement of the thermoelectric efficiency across the different geometries studied in this work (different distances between transversal connections ranging from 220 to 720 nm) while maintaining similar values of electrical conductivity.

The physical phenomena behind the reduction of the thermal conductivity (which reaches values as low as 0.5 W·m^–1^·K^–1^, compared to 2.2 W·m^–1^·K^–1^ for films) and the more than two-fold increase in measured Seebeck coefficient (over −100 μV·K^–1^, reaching even −127 μV·K^–1^, while both thin film and nanowires do not exceed −60 μV·K^–1^) are attributed to the nanostructure of the metamaterial, which can be easily controlled by manipulating the template structure. It is also important to highlight that *zT* for these structures in the in-plane direction only depends on their Seebeck coefficient and the Lorentz number, not on the electrical and thermal conductivities anymore.

This metamaterial overcomes the drawbacks that nanowire structures present (difficult handling when the matrix is dissolved, reduced number of measurement methods to characterize their transport properties). Moreover, this metamaterial appears as a quite attractive alternative to electrodeposited thin films, being easily implemented in actual devices and providing one order of magnitude higher *zT* compared to them.

## Supporting Information Available

Measured compositions of the different samples by EDX, Seebeck coefficients, and electrical and thermal conductivities (Table SI); schematic view of the different preparations of the samples for the measurements (Figure S1); schematic view of the 3D nanowire structure and steady-state solutions of the COMSOL electrical model (Figure S2); heat flux steady-state profile according to the hydrodynamic model for a free-standing 3D network when the temperature gradient is imposed in the in-plane direction (Figure S3); X-ray diffraction spectra of the different samples (Figure S4); and lattice thermal conductivity according to the hydrodynamic heat transport model (Figure S5) (PDF)

## Notes

The authors confirm that the data supporting the findings of this study are available within the article and its Supporting Information.
