# Supplementary material for: 3D Bi2Te3 Interconnected Nanowire Networks to Increase Thermoelectric Efficiency
Source: ACS Appl Energy Mater. 2021 Dec 13;4(12):13556–66. doi: 10.1021/acsaem.1c02129 (PMC9127787; doi:10.1021/acsaem.1c02129)
Supplement: Supplementary file 1 — ae1c02129_si_001.pdf [file ae1c02129_si_001.pdf]

Supporting Information:

3D Bi_2Te_3 interconnected nanowire networks to increase thermoelectric efficiency

Alejandra Ruiz-Clavijo [a], Olga Caballero-Calero [a], Cristina V. Manzano [a], Xavier Maeder [b], Albert Beardo [c], Xavier Cartoixà [d], F. Xavier Álvarez [c], Marisol Martín-González [a, *]

[a] Instituto de Micro y Nanotecnología, IMN-CNM, CSIC (CEI UAM+CSIC) Isaac Newton, 8, E-28760, Tres Cantos, Madrid, Spain

[b] EMPA, Swiss Federal Laboratories for Materials Science and Technology, Laboratory for Mechanics of Materials and Nanostructures, Feuerwerkerstrasse 39, CH-3602 Thun, Switzerland

[c] Departament de Física, Universitat Autònoma de Barcelona. Campus Bellaterra. 08193, Bellaterra. Barcelona

[d] Departament d'Enginyeria Electrònica, Universitat Autònoma de Barcelona. Campus Bellaterra. 08193, Bellaterra. Barcelona

Corresponding author's email[*]: Marisol.martin@csic.es

Table S.I: Composition as found by EDX (with an associated error of 5%), front growth, transport coefficients (electrical conductivity and Seebeck), and thermal conductivity measured in the in-plane direction for the three samples included in this study with different distances between the transversal canals (period, *P*). The thermal conductivity from the model includes the electronic and phononic contributions and the heat flux transfer in the oxide matrix (see methods).

P (nm)	Comp.	Total metamaterial thickness (μm) ± 0.5	Sheet Resis. (Ω·□) ‖c-axis	Resist. (Ω·m) ·10^{-5} ‖c-axis	σ (S·m^{-1}) ·10^{4} ‖c-axis	Seebeck (μV·K^{-1}) ‖c-axis	K_{comp} (W·m^{-1}·K^{-1}) ⊥ c-axis	K_{comp} from the model (W·m^{-1}·K^{-1}) ⊥ c-axis
220	$Bi_{2.0}Te_{3.0}$	11.2	8.6	1.4 ± 0.2	7.1 ± 0.6	- 127 ± 6	0.509 ±0.13	0.508
346	$Bi_{2.0}Te_{3.0}$	11.2	18.1	1.5 ± 0.2	6.6 ± 0.6	- 103 ± 5	0.578 ±0.13	0.593
720	$Bi_{2.0}Te_{3.0}$	12.8	20.9	1.0 ± 0.1	9.8 ± 0.9	- 121 ± 6	0.790 ±0.15	0717

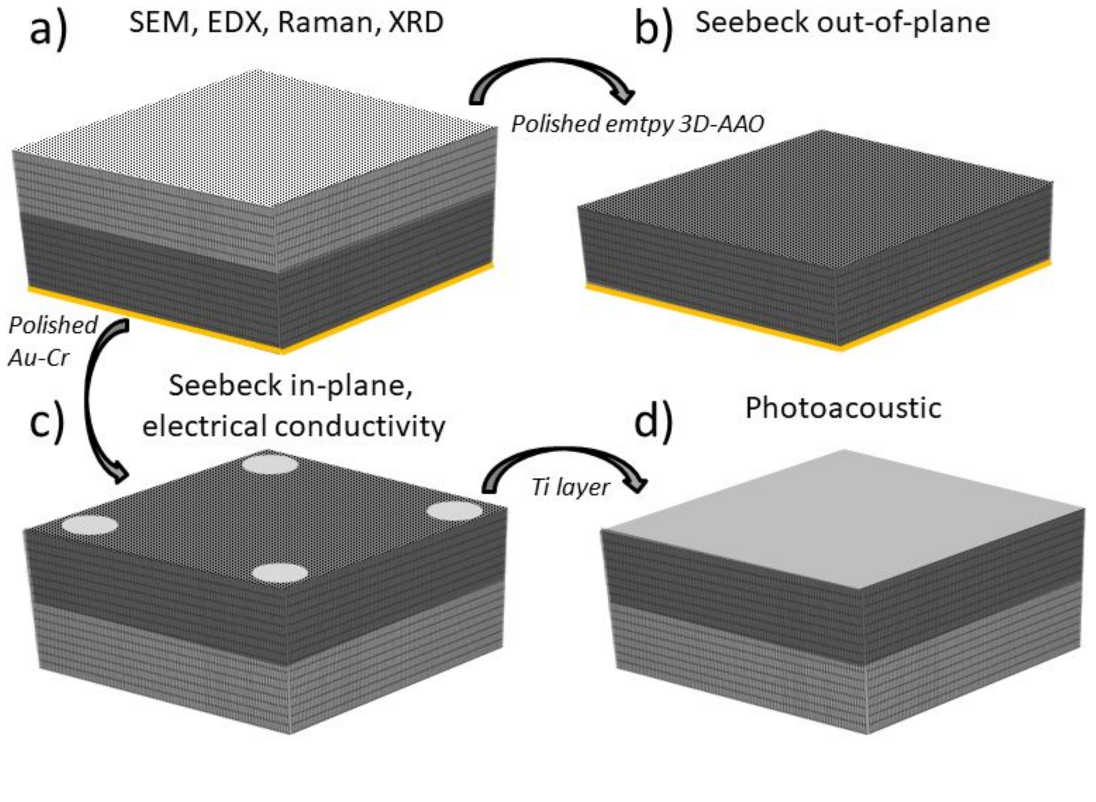

Figure S1: Schematic view of the different preparations of the 3D-Bi_2Te_3 nanowire networks for each of the measurements (not to scale): a) as grown, with the gold and chromium layer, for SEM, Raman, EDX (usually measured in the side of the sample) and XRD; b) after polishing the empty template, keeping the gold –chromium contact at the bottom; c) preparation for in-plane measurement of the Seebeck coefficient and the electrical conductivity. This involves the polishing of the gold-chromium contact and placing 2 (for Seebeck) or 4 (for electrical conductivity measurement) contacts in the surface; d) same preparation than in e) with a 80 nm thick Ti layer evaporated on the surface for photoacoustic measurements.

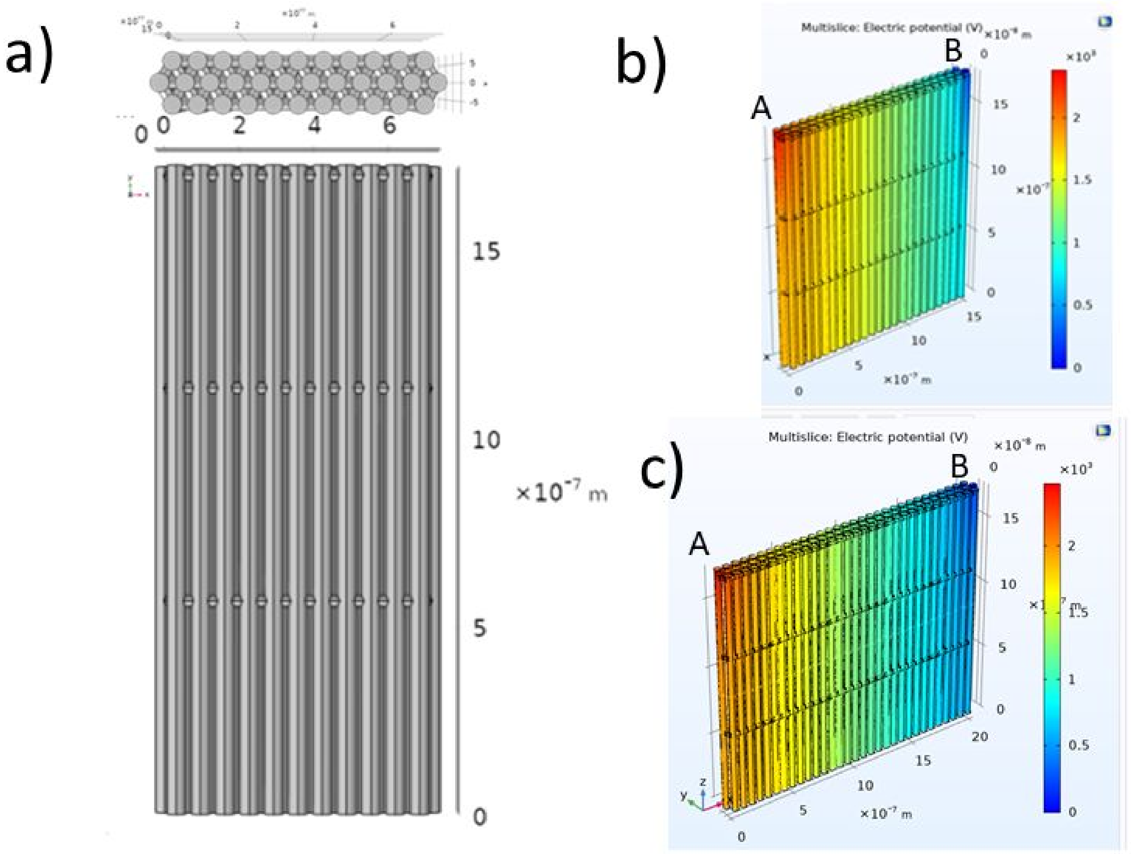

Figure S2: a) top view and side view of the 3D nanowire structure used in the COMSOL model. b) and c) results of the electric potential steady-state solution when the terminals are located at the ends named as A and B.

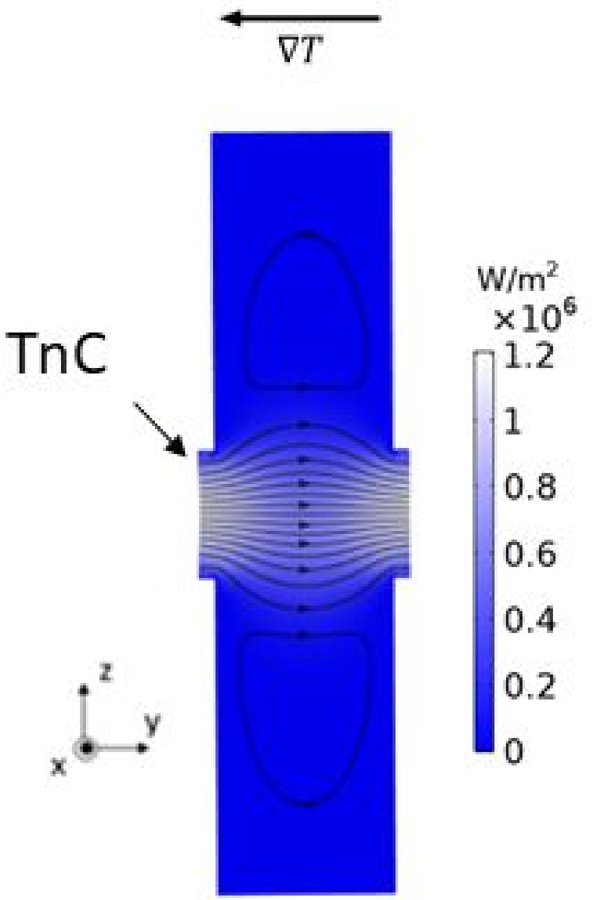

Figure S3: Heat flux steady-state profile and heat flux streamlines according to the hydrodynamic model for a free-standing 3D network when the temperature gradient is imposed along the TnCs direction (in-plane). We obtain the same non-local effects leading to a reduced lattice thermal conductivity that are observed in the out-of-plane direction (Figure 2c). However, in this case, a further reduction is obtained due to the short distance between nanowires, which cause an enhanced inhomogeneity in the heat flux profile.

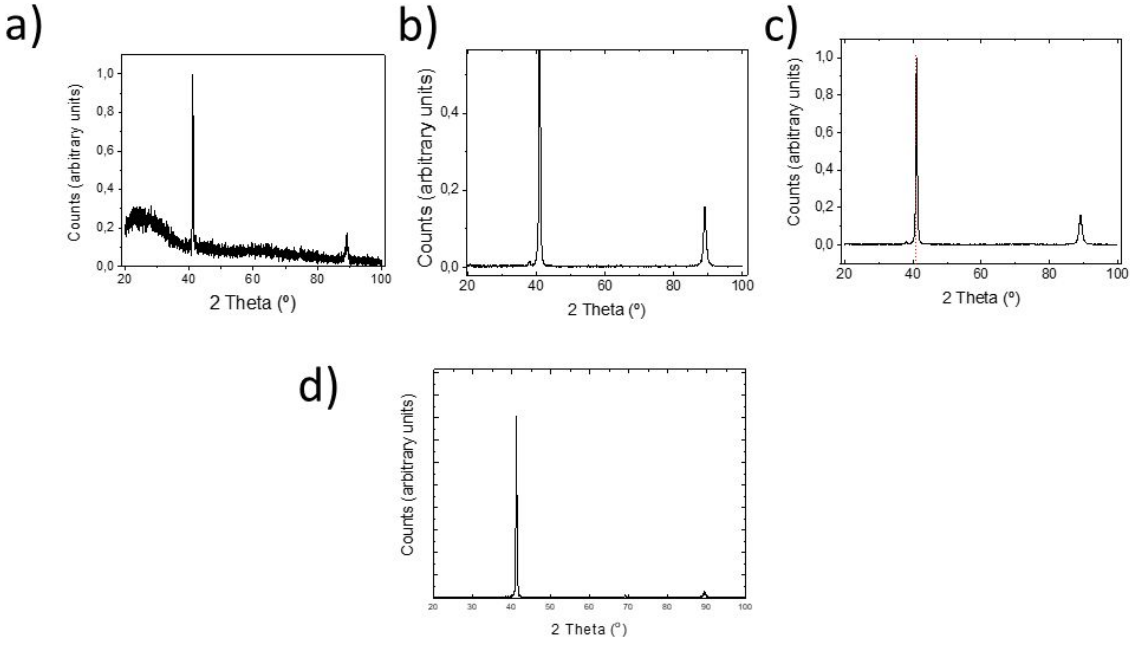

Figure S4: X-Ray Diffraction spectra of the samples with the transversal channels separated (*P*) a) 720, b) 346, and c) 220 nm, and d) of an electrodeposited thin film, with the directions (110) at 41.15°, (220) at 89.28° and, in the case of a), a small peak at 74.81° corresponding to (300), present in the spectra.

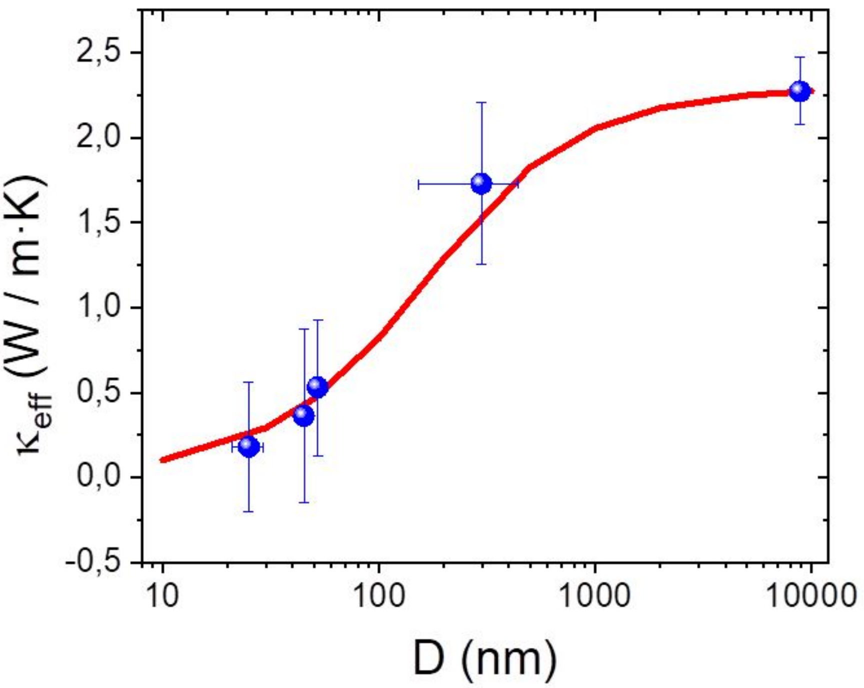

Figure S5: Lattice thermal conductivity according to the hydrodynamic heat transport model (continuous red line) as compared to the experimental data [1] for 1D NWs without the TnCs (blue circles).

References

[1] Rojo, M. M.; Abad, B.; Manzano, C.; Torres, P.; Cartoixà, X.; Alvarez, F.; Gonzalez, M. M., Thermal conductivity of Bi 2 Te 3 nanowires: how size affects phonon scattering. *Nanoscale* **2017,** *9* (20), 6741-6747.
